# Around the Hospitals

**Published:** 1892-07-09

**Authors:** 


### Around the Hospitals.

The **Westminster Hospital** report for 1892 shows a slight increase in the amount of annual subscriptions, but the very reasonable desire to raise £2,000 per annum through this source has not yet been fulfilled, the largest amount thus received in any one year being £1,469. The gross expenditure of the hospital was £14,229 during the past year, to provide which it was necessary, unfortunately, to have recourse to the capital to the extent of £3,700. Repairs of a costly kind were executed during the year, and for this purpose the hospital was closed for six weeks. The percentage of mortality was ·24 less than that of 1890, in spite of the increase of deaths during the months in which influenza was so prevalent. Such a fact is a welcome feature in the year's record of any hospital. We are glad to see that a just tribute is paid to the services of the nursing department, an act of fairness only, which is, unfortunately, too often overlooked in the reports of hospitals.

The **North-Eastern Hospital for Children** is now in full working order. The alterations in the drainage system, for which purpose the hospital had to be closed for some time during the past year, are now completed, and meet all the requirements of modern sanitary science. To the closing of the institution is due the diminution of in and out patients apparent in the report. The Convalescent Homes at Northwold and Cardington, and the Samaritan Fund have been very much appreciated. To the Samaritan Fund £50 was voted from the proceeds of the festival of 1891, at which the Duke of Connaught presided, and at his special request. The new wing, opened recently by the Duchess of Portland, will greatly increase the useful work of the hospital.

The **Louth Dispensary and Hospital** has not been allowed to suffer from the depression of the times which the town is deeply feeling, as is proved by the Annual Report of the Committee. The total income in the past year, including subscriptions, amounted to £772 16s., being £43 16s. 4d. over that of the previous year. A debt of £71 19s. has been reduced to £61 0s. 10d. The Hospital Saturday Fund realised £53 10s., a substantial increase of £28 6s. 2d. on the year before. The Samaritan Fund amounted to £98 12s. 5d., and the sums collected in the churches and chapels came to £124 16s. The twenty beds of the hospital are often all occupied. In the dispensary 700 patients were treated; 2,056 attendances made; and 195 patients visited in their own homes. The only salary received by the medical staff is given to the dispensing surgeon. The Matron, Miss Bradwell, has, during her ten years of office, performed her arduous duties efficiently, without the services of a trained nurse or other trained assistance. Although the hospital stands in an isolated situation, there is no porter, a defect that we trust may be remedied in time. The responsibility incurred by the Matron is far too great injustice to herself or the management. The hospital is a picturesque building prettily situated. The two large wards are very cheerful, having semi-circular balconies at the east end, enclosed with glass.

The **County Infirmary of Carmarthen** shows a period of activity during the past year, and many improvements and repairs to the building have been made. The floors of the largest wards have undergone renovation, and beds have been replaced by those of the best pattern. An empty ward has been entirely furnished with new and improved appliances, and is hitherto to be regarded as an accident ward. The complete repair of the roof is also projected. Donations and subscriptions show a slight falling-off as compared with those of the previous year, but it is to be hoped that sympathy with the efforts of the infirmary will find many new friends for it during the year before us.

